# Endometrioid adenocarcinoma with choriocarcinomatous differentiation: A case report and review of the literature

**DOI:** 10.3892/ol.2013.1431

**Published:** 2013-06-28

**Authors:** MITSUAKI ISHIDA, HIDETOSHI OKABE

**Affiliations:** Department of Clinical Laboratory Medicine and Division of Diagnostic Pathology, Shiga University of Medical Science, Otsu, Shiga 520-2192, Japan

**Keywords:** endometrioid adenocarcinoma, choriocarcinomatous differentiation, chorionic gonadotrophin

## Abstract

A choriocarcinomatous component is rarely present in carcinomas of certain sites and few cases of choriocarcinomatous differentiation in endometrioid adenocarcinoma have been reported. The present study reports a case of endometrioid adenocarcinoma of the uterine corpus with choriocarcinomatous differentiation, and discusses the clinicopathological features of this rare tumor. A 59-year-old post-menopausal female presented with abnormal vaginal bleeding. Magnetic resonance imaging demonstrated a relatively well-circumscribed tumor in the uterine corpus and a total cystectomy was subsequently performed. A histopathological examination revealed two distinct components in the uterine corpus tumor. The first component comprised ~80% of the tumor and was composed of poorly-differentiated endometrioid adenocarcinoma. The remaining component consisted of mononucleated and syncytial giant cells containing rich eosinophilic cytoplasm and large pleomorphic nuclei with coarse chromatin. An immunohistochemical analysis revealed that these syncytial giant cells were positive for β-human chorionic gonadotropin (hCG). Therefore, a diagnosis of endometrioid adenocarcinoma with choriocarcinomatous differentiation was confirmed. The clinicopathological features of nine previously reported cases of this tumor were analyzed in addition to the present case. The majority of the patients were post-menopausal. Endometrial choriocarcinoma may be considered to have a highly aggressive clinical course, since nine of the 10 cases displayed metastases and four patients succumbed to the disease. The pathogenesis of the choriocarcinomatous component is not well understood. However, genetic studies have demonstrated that conventional carcinoma and choriocarcinomatous components share common genetic alterations. The choriocarcinomatous component represents aberrant differentiation of the conventional carcinoma, however, genetic analyses of endometrioid adenocarcinoma with choriocarcinomatous differentiation have not been performed.

## Introduction

Choriocarcinoma is a rare malignant neoplasm composed of mononucleated and multinucleated trophoblasts, mainly arising in the uterus of pregnant females and in the ovaries. A choriocarcinomatous component is rarely present in carcinomas of certain sites, including the lung (1), breast (2), esophagus (3), stomach (4,5), colon (6,7) and urinary system (8). Furthermore, uterine corpus carcinomas with choriocarcinomatous differentiation have rarely been reported (9–17). The present study describes a case of endometrioid adenocarcinoma of the uterine corpus with choriocarcinomatous differentiation and discusses the clinicopathological features of this rare tumor. Written informed consent was obtained from the patient.

## Case report

### Patient

**Figure 1:**
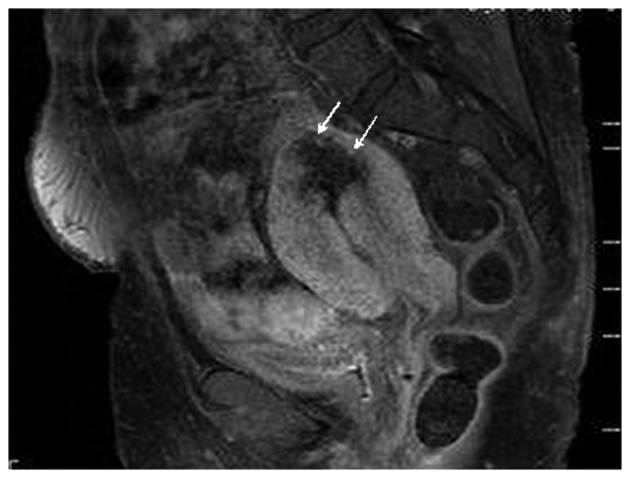
Magnetic resonance imaging showing a relatively well-circumscribed tumor in the fundus of the uterine corpus (arrows).

A 59-year-old post-menopausal female with scleroderma and diabetes mellitus presented with abnormal vaginal bleeding. A physical examination revealed the presence of pyometra and magnetic resonance imaging demonstrated a relatively well-circumscribed tumor, measuring 30 mm in diameter, in the fundus of the uterus (Fig. 1). Swelling of the internal and external iliac and paraaortic lymph nodes was also observed. A clinical diagnosis of a malignant uterine corpus tumor was suspected, and a total cystectomy and bilateral salpingo-oophorectomy were performed, with dissection of the pelvic and paraaortic lymph nodes. Serum β-human chorionic gonadotropin (hCG) levels were not measured.

### Materials and methods

Formalin-fixed, paraffin-embedded tissue blocks were cut into 3-μm thick sections, then deparaffinized and rehydrated. Each section was stained with hematoxylin and eosin and used for immunostaining. Immunohistochemical analyses were performed using an autostainer (Benchmark XT system; Ventana Medical System, Tucson, AZ, USA) according to the manufacturer’s instructions. The following primary antibodies were used: Mouse monoclonal antibody against CA125 (Ov185:1; Novocastra Laboratories, Ltd., Newcastle upon Tyne, UK), mouse monoclonal antibody against cytokeratin (AE1/AE3; DAKO Cytomation, Glostrup, Denmark) and rabbit polyclonal antibody against human β-hCG (Novocastra).

### Histopathological findings

**Figure 2:**
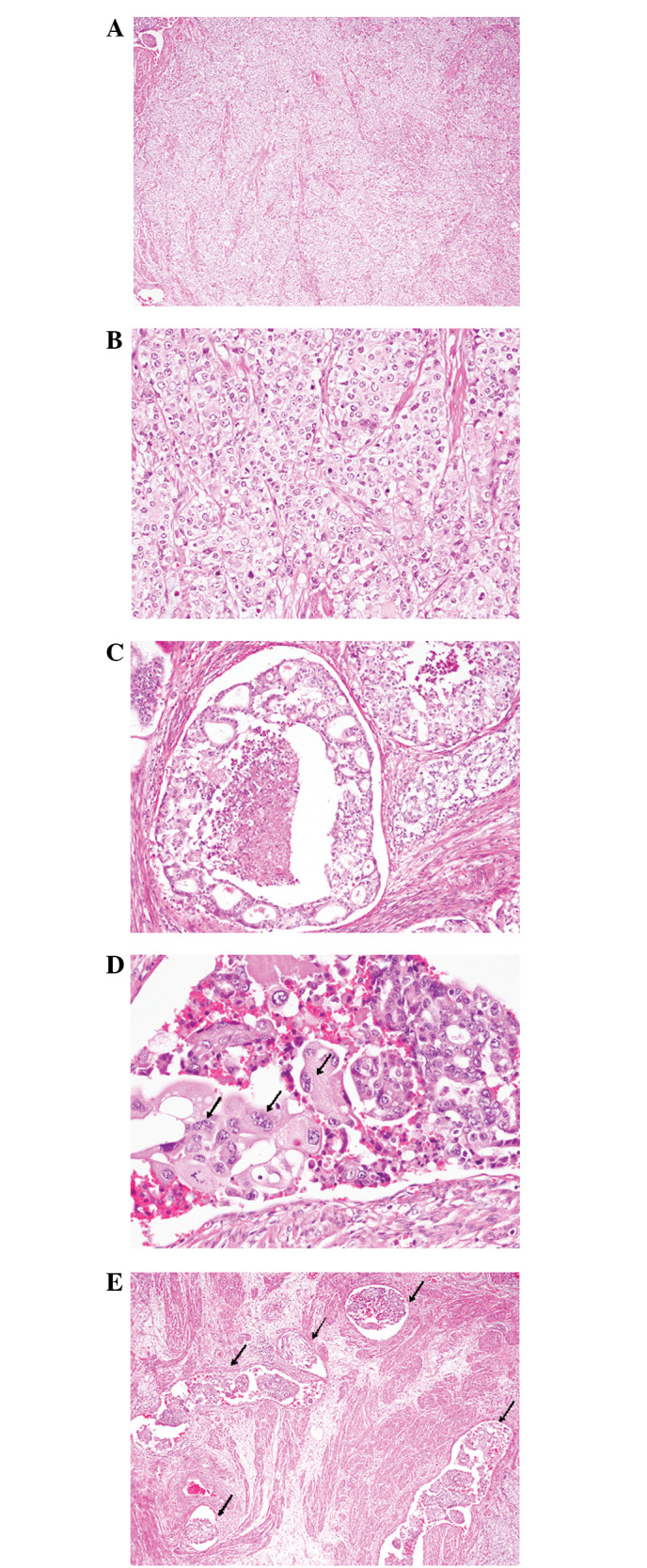
Histopathological findings of the uterine corpus tumor. (A) Poorly-differentiated endometrioid adenocarcinoma component. Proliferation of sheets or variable-sized nests of atypical epithelial cells. (hematoxylin and eosin; magnification, ×40). (B) Atypical epithelial cells contain a relatively rich, marginally eosinophilic cytoplasm and large nuclei with coarse chromatin and small nucleoli (hematoxylin and eosin; magnification, ×200). (C) Glandular differentiation showing a cribriform structure with central necrosis (hematoxylin and eosin; magnification ×100). (D) Choriocarcinomatous component. Syncytial giant cells are scattered (arrows; hematoxylin and eosin; magnification, ×200). (E) Vascular and lymphatic invasions are prominent (arrows; hematoxylin and eosin; magnification, ×40).

The uterine corpus tumor consisted of two distinct histopathological components. The first component comprised ~80% of the tumor and was composed of a poorly-differentiated adenocarcinoma with extensive hemorrhage and necrosis. This area involved a proliferation of sheets or variable-sized nests of atypical epithelial cells containing large oval nuclei with coarse chromatin and small nucleoli (Fig 2A and B). These tumor cells contained a relatively rich, marginally eosinophilic cytoplasm, but no intracytoplasmic mucin was observed (Fig. 2B). Mitotic figures were frequently observed (34/10 high-power fields). Focal glandular differentiation showing cribriform glands with central necrosis was present (Fig. 2C), however, no squamous differentiation was noted. Accordingly, this component was considered to be an endometrioid adenocarcinoma. The remaining component consisted of mononucleated and syncytial-like giant cells, with a rich eosinophilic cytoplasm and large pleomorphic nuclei with coarse chromatin (Fig. 2D). There was a transition between the endometrioid adenocarcinoma and choriocarcinomatous components (Fig. 2D). A number of lymphatic and vascular invasions were noted (Fig. 2E). The tumor had invaded deeply into the entire layer of the uterine corpus wall and serosal invasion was also noted. However, no parametrial or vaginal invasion was observed. The internal and external iliac and paraaortic lymph nodes exhibited metastatic carcinomas accompanying each of the components.

### Immunohistochemical findings

**Figure 3:**
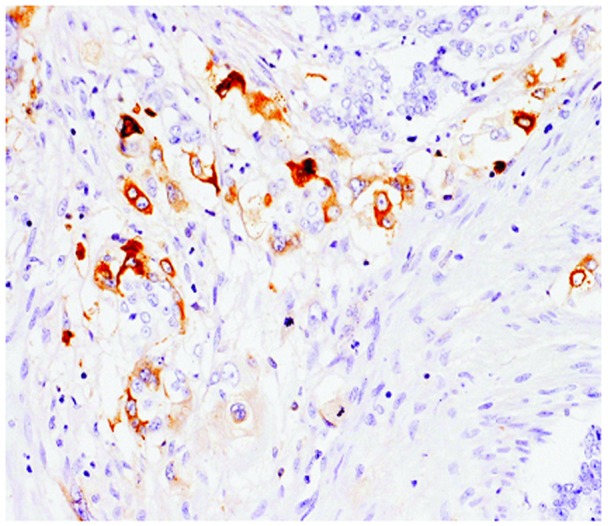
Immunohistochemical features of the uterine corpus tumor. β-human chorionic gonadotrophin (hGC) is expressed in the syncytial giant cells (x200).

Cytokeratin (AE1/AE3) was expressed in the endometrioid adenocarcinoma and choriocarcinomatous components. CA125 was expressed in the endometrioid adenocarcinoma component, but not in the choriocarcinomatous component. β-hCG was expressed in the choriocarcinomatous component, particularly in the syncytial giant cells (Fig. 3), but not in the endometrial carcinoma component. Metastatic lesions of the lymph nodes showed similar immunohistochemical features to the primary tumor and β-hCG-positive syncytial giant cells were scattered amongst the metastatic lesions.

According to the histopathological and immunohistochemical features, an ultimate diagnosis of endometrioid adenocarcinoma with choriocarcinomatous differentiation was made [pIIIC2; International Federation of Gynecology and Obstetrics (FIGO)].

## Discussion

The present study describes a case of endometrioid adenocarcinoma of the uterine corpus with choriocarcinomatous differentiation. Civantos and Rywlin first reported a case of uterine corpus carcinoma (serous papillary adenocarcinoma) with choriocarcinomatous differentiation in 1972 (16). Subsequently, Savage *et al* reported the first case of endometrioid adenocarcinoma of the uterine corpus with choriocarcinomatous differentiation in 1987 (9). Since then, few uterine corpus adenocarcinomas with choriocarcinomatous differentiation have been reported (10–15). The most common histopathological subtype of the carcinomatous component is endometrioid adenocarcinoma (9–15), as seen in the present case. Serous papillary adenocarcinoma (16,17), clear cell adenocarcinoma (18) and carcinosarcoma with choriocarcinomatous differentiation (19,20) have also been documented. In addition, uterine cervical adenocarcinoma with choriocarcinomatous differentiation has also been reported (21).

Table I summarizes the clinicopathological features of nine previously reported cases of endometrioid adenocarcinoma of the uterine corpus with choriocarcinomatous differentiation, in addition to the present case. The median age of the patients was 62.3 years (range, 42–83 years) and the majority were post-menopausal females, with the exception of the case reported by Akbulut *et al*, in which the patient was a 42-year-old premenopausal female (15). Abnormal vaginal bleeding and abdominal pain were the main presenting symptoms (14,15). Endometrial choriocarcinoma may be considered to have a highly aggressive clinical course, since nine of the 10 cases studied showed metastases, with the common metastatic sites being the lung, liver and lymph nodes, while four patients succumbed to the disease (Table I). The histopathological features of the metastatic sites were variable; two cases, including the present case, shared the same features as the primary site (adenocarcinoma with choriocarcinomatous components) and two cases only exhibited the choriocarcinoma component (Table I).

Although the pathogenesis of the choriocarcinomatous component in non-gestational tumors is not well understood, studies suggest that the choriocarcinomatous component probably represents heterotopic or aberrant differentiation of the conventional carcinoma components, rather than the malignant transformation of germ cells (7,22). Zetll *et al* reported a case of urothelial carcinoma with a choriocarcinomatous component and analyzed the comparative genomic hybridization of the two components (22). The study clearly demonstrated that the components shared losses of chromosomes 9 and 17p, which were characteristic genetic alterations of urothelial carcinoma, and that the choriocarcinomatous components acquired additional chromosomal losses and gains, mostly associated with poorly-differentiated urothelial carcinoma (22). The results suggest a close genetic association between urothelial carcinoma and the choriocarcinomatous component. Furthermore, Verbeek *et al* reported a case of rectal adenocarcinoma with choriocarcinomatous components and identified genetic changes that are characteristic of colorectal adenocarcinoma (losses of chromosomes 8p and 18q and gains of 5p and 20q) in the two components, providing evidence for a common origin (7). According to these results, the two histological components of endometrioid adenocarcinoma with choriocarcinomatous differentiation may share a common genetic origin, however, genetic analyses of this rare tumor have not been performed.

In conclusion, the present study describes the 10th documented case of endometrioid adenocarcinoma of the uterine corpus with choriocarcinomatous differentiation. The clinicopathological analyses revealed that this rare tumor has a highly aggressive clinical course, with a high incidence of metastases and a high mortality rate. Therefore, identifying the choriocarcinomatous component in endometrioid adenocarcinoma is essential for establishing an adequate therapeutic strategy.

## Figures and Tables

**Table I:** Clinicopathological features of endometrioid adenocarcinoma with choriocarcinomatous differentiation.

Case no.	Age (years)	Histopathology of coexisting tumor	Metastases or invasion	Histopathology at metastatic sites	Outcome, months	Reference
1	70	WD	Brain, lung, liver, kidneys	Choriocarcinoma	DOD, 14	9
2	78	PD	Pelvic lymph nodes	NA	DOD, 1.5	10
3	48	PD	Lungs	NA	AWD, 2	10
4	63	Adenocarcinoma	Lungs, liver, peritoneum	Same as primary site	DOD, 14	10
5	83	MD	Lungs	NA	AWD, 1	11
6	68	PD, clear cell and serous papillary	Pelvic lymph nodes	Serous papillary adenocarcinoma	NED, 16	12
7	54	MD	Retroperitoneum	Adenocarcinoma	DOD, 24	13
8	58	WD	Vaginal cuff	Choriocarcinoma	NED, 36	14
9	42	MD	None		NED, 6	15
Present case	59	PD	Iliac and paraaortic lymph nodes	Same as primary site	NED, 2	

WD, well-differentiated adenocarcinoma; PD, poorly-differentiated adenocarcinoma; MD, moderately-differentiated adenocarcinoma; NA, not available; AWD, alive with disease; DOD, died of disease; NED, no evidence of disease.
